# Neddylation of protein, a new strategy of protein post-translational modification for targeted treatment of central nervous system diseases

**DOI:** 10.3389/fnins.2024.1467562

**Published:** 2024-11-05

**Authors:** Qian Wu, Ziang Geng, Jun Lu, Shisong Wang, Zhongxue Yu, Siqi Wang, Xiaolin Ren, Shu Guan, Tiancong Liu, Chen Zhu

**Affiliations:** ^1^Department of Neurology, The First Hospital of China Medical University, Shenyang, Liaoning, China; ^2^Department of Neurosurgery, Shengjing Hospital of China Medical University, Shenyang, Liaoning, China; ^3^Department of Neurosurgery, The First Hospital of China Medical University, Shenyang, Liaoning, China; ^4^Department of Cardiovascular Ultrasound, The First Hospital of China Medical University, Shenyang, Liaoning, China; ^5^Department of Radiation Oncology, The First Hospital of China Medical University, Shenyang, Liaoning, China; ^6^Department of Neurosurgery, Shenyang Red Cross Hospital, Shenyang, Liaoning, China; ^7^Department of Surgical Oncology and Breast Surgery, The First Hospital of China Medical University, Shenyang, Liaoning, China; ^8^Department of Otolaryngology, Shengjing Hospital of China Medical University, Shenyang, Liaoning, China

**Keywords:** neddylation, central nervous system, neurology, glioma, targeted therapy

## Abstract

Neddylation, a type of protein post-translational modification that links the ubiquitin-like protein NEDD8 to substrate proteins, can be involved in various significant cellular processes and generate multiple biological effects. Currently, the best-characterized substrates of neddylation are the Cullin protein family, which is the core subunit of the Cullin-RING E3 ubiquitin ligase complex and controls many important biological processes by promoting ubiquitination and subsequent degradation of various key regulatory proteins. The normal or abnormal process of protein neddylation in the central nervous system can lead to a series of occurrences of normal functions and the development of diseases, providing an attractive, reasonable, and effective targeted therapeutic strategy. Therefore, this study reviews the phenomenon of neddylation in the central nervous system and summarizes the corresponding substrates. Finally, we provide a detailed description of neddylation involved in CNS diseases and treatment methods that may be used to regulate neddylation for the treatment of related diseases.

## 1 Introduction of protein neddylation

**Figure 1:**
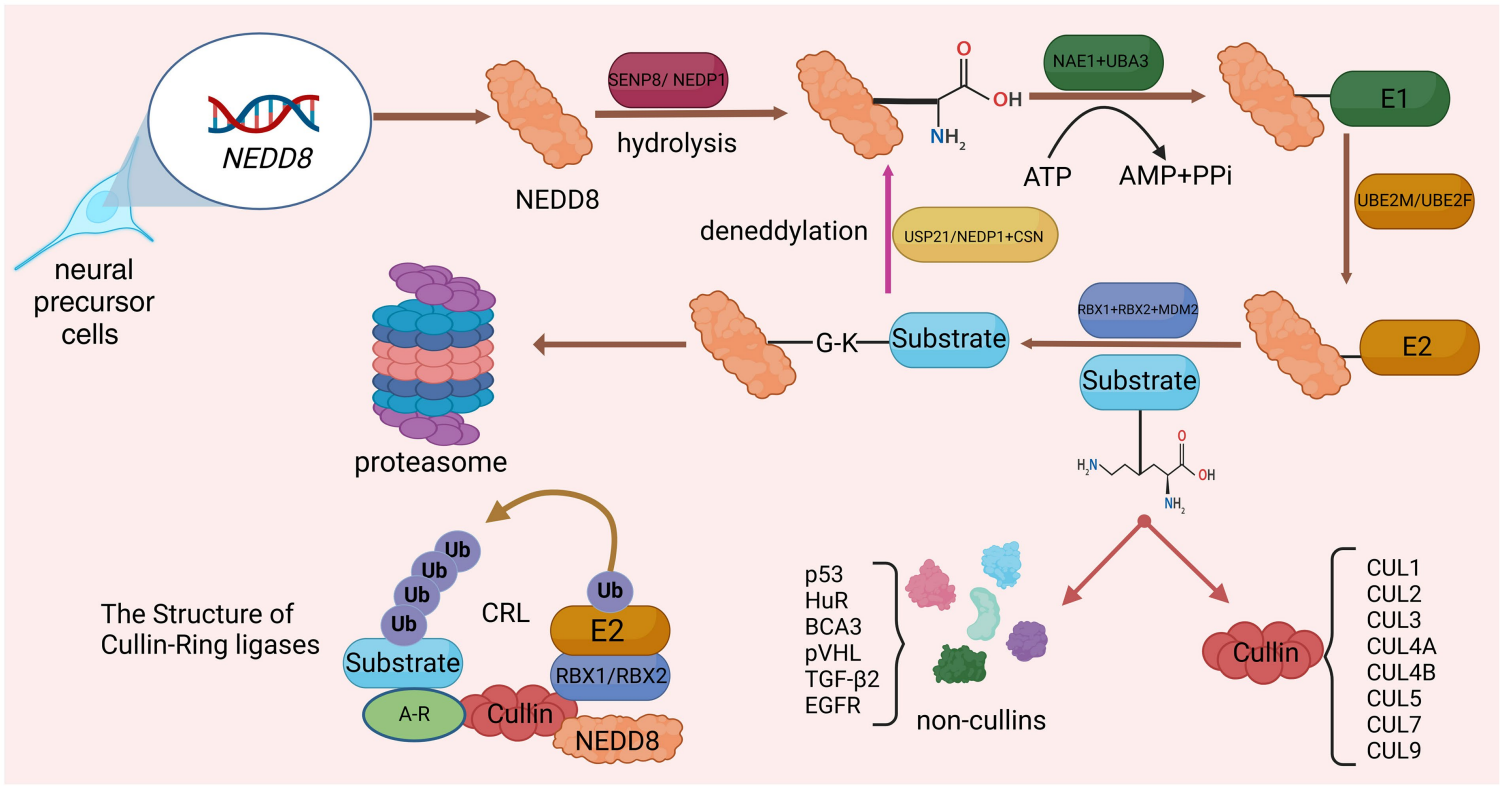
Detailed processes of neddylation and deneddylation and related biological effects. After activation by SENP8, the lysine residue at the C-terminus of NEDD8 precursor is exposed. Subsequently, through the catalysis of E1, E2, and E3, NEDD8 was connected to the substrate through four steps: activation, conjugation, ligation, and neddylation. The substrate that has been neddylated can participate in biological processes such as protoasome construction.

Neddylation is a new protein post-translational modification (PTM) process discovered in recent years, which was first reported by Tetsu Kamitani and other researchers (Kamitani et al., 1997). Up to now, researchers have found that neddylation is widely present in eukaryotic organisms and is involved in regulating a variety of biological processes, such as cell cycle, signal transduction, and immune response. The core process of neddylation is mediated by Neural Precursor Cell Expressed Developmentally Downregulated Protein 8 (NEDD8), which connects the glycine residue at the C terminus of the NEDD8 protein to the lysine residue of the substrate, affecting protein stability and subcellular localization and activity, thereby regulating various biological processes (Duda et al., 2008). Similar to ubiquitination, neddylation is a highly dynamic four-step enzymatic cascade. NEDD8 gene expression produces NEDD8 precursor, which is processed by SUMO Peptidase Family Member, NEDD8 Specific (SENP8/NEDP1), exposing the C-terminal glycine of NEDD8 (Enchev et al., 2015). After proteolysis, the NEDD8 E1 activating enzyme (E1) consisting of NEDD8 Activating Enzyme E1 Subunit 1 (NAE1) and Ubiquitin Like Modifier Activating Enzyme 3 (UBA3) promotes NEDD8 activation in an adenosine 5′-triphosphate (ATP)-dependent manner, activating and generating a high-energy intermediate (Walden et al., 2003). Subsequently, Ubiquitin Conjugating Enzyme E2 M (UBE2M) or Ubiquitin Conjugating Enzyme E2 F (UBE2F), acting as NEDD8 E2 conjugating enzyme (E2), accepts NEDD8 and transfers it to the NEDD8 E3 ligase (E3), ensuring that activated NEDD8 interacts with its specific coupling between target proteins (Huang et al., 2005). E3s that have been discovered in research include Ring-Box 1 (RBX1; Huang et al., 2009), Ring-Box 2 (RBX2; Monda et al., 2013) and Murine Double Minute 2 Proto-Oncogene (MDM2; Bailly et al., 2016). Finally, similar to ubiquitination, E3 connects the conserved C-terminal glycine residue of NEDD8 and the lysine residue of the substrate to complete the process of neddylation modification of the substrate. Deneddylation is mediated and completed by NEDP1 and COP9 Signalosome (CSN; Tian et al., 2023). In addition, Ubiquitin Specific Peptidase 21 (USP21) can also participate in deneddylation (Yu et al., 2018; Figure 1).

The most characteristic substrate for neddylation is the cullin protein family (CUL), which is the core subunit of the Cullin-RING E3 ubiquitin ligase complex (CRL). CRL, also known as the NEDD8 ubiquitin ligase complex, includes CUL1, CUL2, CUL3, CUL4A, CUL4B, CUL5, CUL7, and CUL9 (Boh et al., 2011). The binding of NEDD8 to the C-terminal lysine residue of CUL will change the conformation of CUL, causing CRL to activate and regulate protein ubiquitination and degradation, and ultimately selectively degrade key proteins through the ubiquitin-protease system (Yamoah et al., 2008). Other neddylated substrate proteins include p53 (Liu et al., 2023), HuR (de Sousa et al., 2015), BCA3 (Gao et al., 2006), pVHL (Wolf et al., 2020), TGF-β2 (Zuo et al., 2013), and EGFR (Noronha et al., 2022). The process of neddylation modification of NEDD8 will affect the progress of various life activities. Studies have shown that during proteotoxic stress, neddylation modification of proteins promotes the aggregation of nuclear proteins (mainly ribosomal proteins), thereby protecting the nuclear ubiquitin-proteasome system from stress-induced dysfunction and ensuring protein quality control networks carry out protein repair and refolding via molecular chaperones (Xirodimas et al., 2008; Figure 1).

Neddylation is critical in maintaining all aspects of the development of cells, organs, and organ functions, and interference with the neddylation pathway can lead to the occurrence of a series of dysfunctions and diseases, especially in the central nervous system (CNS). Neddylation is involved in the development process of CNS and is related to processes such as neuronal apoptosis and neuronal autophagy (Balasubramaniam et al., 2019). Studies have shown that the process of neddylation increases during brain development in mice within 2 weeks of birth and remains present throughout adulthood (Vogl et al., 2015). There is evidence that neddylation is involved in the process of neuronogenesis, by regulating the maturation and stability of neuronal dendritic spines, thereby promoting neurotransmitter transmission (Joo et al., 2011). Recently, neddylation has been shown to be required for the maturation of neuronal postsynaptic density protein 95, thereby increasing synaptic conduction excitability (Vogl et al., 2020). In addition, amyloid precursor protein (APP) downregulates the Wnt/β-catenin signaling pathway by excessively activating neddylation, thereby inducing neuronal apoptosis and ultimately affecting cortical development (Chen et al., 2003). The dysfunction of the interaction between APP and its interacting protein Amyloid Beta Precursor Protein-Binding Protein 1 (APPBP1) may be one of the causes of inducing certain neurological diseases. Neddylation activated by APPBP1 downregulates EGFR and APP signaling, thereby affecting neuronal differentiation potential (Zhang et al., 2020). Neddylation is also involved in the occurrence and development of multiple CNS diseases, including intracranial tumors (Qi et al., 2024), cerebrovascular diseases (Yu et al., 2022), central nervous system demyelinating diseases (Kim et al., 2021), movement disorder diseases (Saurat et al., 2024), epilepsy (Chen et al., 2021), neurological degenerative diseases (Kassouf et al., 2023) and neurological genetic diseases (Sardina et al., 2024), etc. These diseases are important causes of disability or death in patients. Therefore, there is an urgent need to clarify the mechanism and role of neddylation in the CNS and to find possible methods for targeted therapy. Therefore, this review aims to comprehensively and systematically elucidate the regulation of protein neddylation modifications and their role in CNS development and disease occurrence and development and to evaluate neddylation as a therapeutic target for CNS diseases. We hope that this study can provide new insights into future research directions.

## 2 Description for protein neddylation mechanism in CNS

### 2.1 NEDD8 E1 activating enzyme induces the activation of NEDD8 precursor

NEDD8 was first cloned from the mouse brain in 1993 and was confirmed to be widely expressed in adult tissues (Kim et al., 2013). The NEDD8 precursor protein produced by NEDD8 gene expression consists of 81 amino acids and undergoes proteolytic processing before acting on the target protein, exposing the 76th glycine residue at the C-terminus. It has been reported that enzymes involved in processing NEDD8 precursors include SENP8 and Ubiquitin C-Terminal Hydrolase L3 (UCHL3; Frickel et al., 2007). Following the proteolytic process, E1 promotes NEDD8 activation in an ATP-dependent manner. E1 is a heterodimer composed of NAE1 and UBA3. Among them, NAE1, also known as APPBP1, constitutes the N-terminus of E1, and UBA3 serves as another subunit corresponding to the C-terminus of E1 (Miles et al., 2015). E1 contains two different active sites, the adenylate activation site located in UBA3 and the cysteine transsulfation domain composed of two subunits. During the E1 catalytic stage, E1 binds NEDD8 and ATP. In the presence of magnesium ions, ATP releases inorganic pyrophosphate and occupies the adenylate activation site of UBA3, thereby forming a high-energy AMP-NEDD8 intermediate (Souphron et al., 2008). Subsequently, E1 catalyzes the reaction of cysteine with the high-energy AMP-NEDD8 intermediate, forming a thioester bond between E1 and NEDD8, and releasing AMP (Cappadocia and Lima, 2018). Finally, the NEDD8-E1 intermediate binds NEDD8 and ATP again, releasing the phosphate group in the presence of magnesium ions, thereby catalyzing the formation of the NEDD8-E1-NEDD8-AMP complex (An and Statsyuk, 2016). In this process, UBA3 is essential for the activation of NEDD8. However, as another subunit of the E1 heterodimer, APPBP1 is not a necessary group for the NEDD8 activation process, but instead serves as a scaffolding protein that accelerates activation kinetics. The activity of the APPBP1-ATP complex contributes to the activation of NEDD8. Furthermore, the stability of the APPBP1-UBA3-NEDD8 complex may depend on the interaction between APPBP1 and UBA3 (Malik-Chaudhry et al., 2018).

An electrophysiological study found that inhibition of the neddylation process caused by NAE1 deletion reduces the stability of the voltage-gated channel α subunit Nav1.1, thereby reducing the excitability of parvalbumin-positive interneuron (PVIN) and increasing the activity of pyramidal neurons (PyN; Chen et al., 2021). Studies have shown that NAE1 mutations can cause damage to the developing mouse cerebral cortex. Specifically, NAE1 mutations in mature PyNs reduce the number of axons and inhibit the formation of excitatory postsynaptic potentials, eventually leading to impairment of the mouse’s learning and memory abilities (Zhang et al., 2020). In addition, primary neurons from rats overexpressing APPBP1 showed significant differences in the number of apoptotic nuclei compared with controls. Subsequent studies confirmed that the interaction between APP and APPBP1 can induce neuronal apoptosis by activating the neddylation pathway and promote neuronal entry into the cell cycle (Chen et al., 2003). Since neurons are not mitotic cells, once they enter the cell cycle, apoptosis is triggered, which may ultimately result in DNA damage and endogenous apoptosis. In addition, activation of the neddylation process promotes the degradation of β-catenin, leading to the loss of intercellular adhesion and making neurons more susceptible to apoptosis (Chen, 2004). As the precursor of amyloid beta (Aβ) protein, APP exists on the surface of neurons and is cleaved by β and γ secretases to produce Aβ and APP intracellular cleavage domains, both of which jointly induce apoptosis (Kinoshita et al., 2002). As a related protein of APP, the neddylation process activated by APPBP1 can weaken the conduction of the EGFR signaling pathway. The cell cycle is inhibited due to the withdrawal of growth factors, which may be one of the reasons for the induction of neural progenitor cell differentiation (Seshacharyulu et al., 2012). Previous studies confirmed that APPBP1 drives the cell cycle at the S-M checkpoint. This function arises from the NEDD8-mediated neddylation pathway, which suggests that neddylation contributes to neuronal division and proliferation (Joo et al., 2011; Table 1).

**Table 1:** Mechanism of protein neddylation in CNS.

Process of neddylation	Key enzyme	Representative enzyme molecule	Effects of neddylation in CNS
1. Activation of NEDD8 Protein	NEDD8 E1 Activating Enzyme	NAE1-UBA3, APPBP1-UBA3	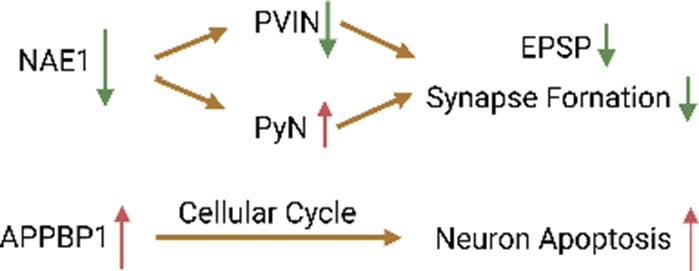
2. Conjugation of NEDD8 Protein	NEDD8 E2 Conjugating Enzyme	UBE2M, UBE2F	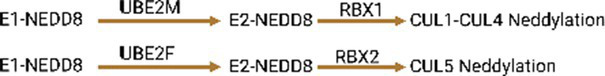
3. Ligation of NEDD8 Protein	NEDD8 E3 Ligase	RBX1, RBX2, MDM2, Rapsyn	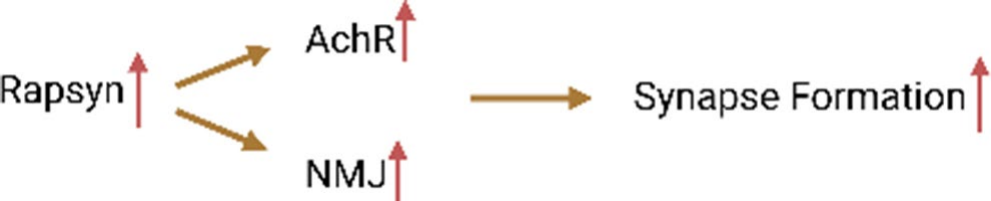
4. Deneddylation	—	CSN, NEDP1	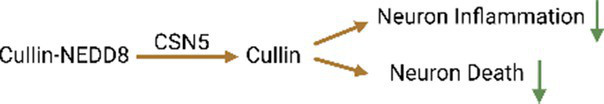

### 2.2 NEDD8 E2 conjugating enzyme mediates formation of NEDD8-E2 complex

In the transfer stage catalyzed by E2, E2 approaches the NEDD8-E1-NEDD8-AMP complex and transfers NEDD8 from the cysteine active site of E1 to the cysteine active site of E2 through a transthioesterification reaction, forming the E2-NEDD8 complex (Huang et al., 2008). In eukaryotic cells, two structurally similar E2s have been discovered, namely the well-studied UBE2M (Gong and Yeh, 1999) and the less-studied UBE2F (Huang et al., 2009). Currently, studies have shown that multiple inhibitors targeting the interaction between UBE2M and Defective In Cullin Neddylation 1 (DCN1) show certain anti-tumor effects (Zhou et al., 2023). Specifically, UBE2M and UBE2F bind to the ubiquitin folding domain of NAE1 and the UBA3 hydrophobic groove through the core domain and N-terminal sequence, respectively, (Huang et al., 2004). Acetylation of the N-terminal methionine residue occurs in both E2s, which facilitates their binding to the PONY domain of Defective In Cullin Neddylation Protein 1-Like Protein 1 (DCUN1D1), thereby increasing cullin family neddylation efficiency of the process (Monda et al., 2013). These two NEDD8-conjugating enzymes appear to have distinct and prominent effects on different protein substrates. For example, UBE2M pairs with RBX1 to mediate neddylation of CUL1-CUL4, while UBC2F interacts with RBX2/SAG to specifically regulate CUL5 neddylation (Kwon et al., 2004). Existing evidence shows that UBE2M plays a dominant role in neddylation modifications, because overexpression of UBE2M itself can induce neddylation modifications, while silencing UBE2M will weaken neddylation modifications (Li et al., 2019). Interestingly, a recent study revealed a cross-talk between two NEDD8-conjugating enzymes, whereby UBE2M acts as an E2 to activate CRL3 to control UBE2F levels under stress-free conditions. Under stress stimulation, UBE2M acts as Parkin’s ubiquitinase 2, promoting the ubiquitination and degradation of UBE2F, leading to the inactivation of CRL5 (Zhou et al., 2018; Table 1).

### 2.3 NEDD8 E3 ligase promotes the ligation of NEDD8 and substrate

In the substrate neddylation modification stage, the NEDD8-E2 complex chemotaxis toward E3 and connects to it to prepare for the completion of the subsequent substrate neddylation process. In contrast to the above-mentioned E1 and E2, the identity of the true specific E3 remains unclear. At present, it has been found that E3s with clear functions all contain RING domains, and common E3s include RBX1, RBX2, and MDM2 (Bao et al., 2023). In addition, Cbl Proto-Oncogene (CBL; Oved et al., 2006), F-Box Protein 11 (FBXO11; Jansen et al., 2019), MNAT1 Component Of CDK Activating Kinase (MNAT1; Rabut et al., 2011), Tripartite Motif Containing 40 (TRIM40; Noguchi et al., 2011), Ring Finger Protein 168 (RNF168; Li et al., 2014) and Ring Finger Protein 111 (RNF111; Ho et al., 2014) also have E3 functions. Interestingly, DCN1 can also act as a NEDD8 ligase, although its structure does not contain the classic RING domain (Lin et al., 2017). Some researchers have proposed that DCN1-5 are E3s that mediate the neddylation process of the Cullin family and act as independent effectors in the process. However, some researchers believe that the DCN family only acts as a cofactor for other E3s (Wu et al., 2011). The classical E3 ligases RBX1 and RBX2 interact with UBE3M and UBE2F, respectively, so that NEDD8 binds to CRL family members (Scott et al., 2014).

One of the E3 ligases, receptor-associated protein at synapse (Rapsyn), serves as a classic scaffolding protein that can anchor AChR to the synaptic cytoskeleton. Rapsyn plays an important role in the formation of acetylcholine receptor (AChR) clustering and neuromuscular junction (NMJ). This is the guarantee of neurotransmitter delivery across the postsynaptic membrane. The researchers found that the RING domain of Rapsyn contains E3 ligase activity. Mutation of the RING domain that abolishes the enzyme activity inhibits Rapsyn- as well as agrin-induced AChR clustering in heterologous and muscle cells. Rapsyn acts as an E3 to induce AChR clustering and NMJ formation, and participates in regulating the neddylation process of AChR (Li et al., 2016). This suggests that neddylation may be a novel mechanism in neural development including synapse formation. In addition, Parkin is also a RING/HECK hybrid E3, with a ubiquitin-like domain at the N terminus and a RING-IBR-RING domain at the C terminus (Wenzel et al., 2011). Parkin and synphilin-1 were also up-regulated with increasing degree of neddylation in a dose-dependent manner. This indicates that NEDD8 enhances Parkin activity (Um et al., 2012). The E3 ligase complex formed by Parkin, PINK1 and DJ-1 promotes the degradation of misfolded or unfolded proteins (Xiong et al., 2009; Table 1).

### 2.4 Nedylation of substrate proteins involve in various cellular processes especially in CNS

To date, the best characterized neddylation substrates are the Cullin family proteins (CUL), including CUL1, CUL2, CUL3, CUL4A, CUL4B, CUL5, CUL7, and CUL9 (Boh et al., 2011). Cullin serves as a scaffold component and is combined with other components, including linkers, substrate receptors, and RING domains, to form Cullin-RING ligase (CRL). Cullin’s neddylation modification is critical for CRL to produce the correct function. Cullin’s neddylation modification can enhance the activity of CRL, which contributes to cell cycle progression and cell survival (Huang et al., 2023). Cullin’s neddylation modification is also called the classic neddylation modification process. However, an atypical neddylation modification process occurs when the substrate protein covalently bound to NEDD8 is not a Cullin molecule. In recent years, more and more non-Cullin substrate proteins have been reported, such as p53, pVHL, TGF-β, and EGFR. The direct biological effects of protein neddylation include three main parts: conformational change, precluding certain interaction and providing a novel binding surface. CUL4 is a member of the Cullin protein family and regulates the autophagy process of cells through neddylation modification. WD Repeat Domain, Phosphoinositide Interacting 2 (WIPI2) is an autophagy-related protein and a direct substrate of CRL4, participating in autophagosome biogenesis. During mitosis induction, CRL4 is activated through neddylation modification and recruits WIPI2 through Damage Specific DNA Binding Protein 1 (DDB1), leading to multiple ubiquitination and proteasomal degradation of WIPI2 and inhibiting autophagy. During cell mitosis, WIPI2 protein levels and autophagy levels can be rescued by knocking down CRL4 or using MLN4924, a selective NEDD8 activating enzyme inhibitor. Moreover, restoration of WIPI2 rescues autophagy during mitosis and leads to mitotic slippage and cell senescence (Lu et al., 2019). Previous studies have shown that Discs Large MAGUK Scaffold Protein 4 (DLG4/ PSD95) is also one of the substrates of the neddylation process, and the neddylation of DLG4 has Helps the stability of the spine (Liang et al., 2022).

The covalent binding of NEDD8 to substrate proteins can be removed through the deneddylation process catalyzed by some deneddylation enzymes, including CSN, NEDP1, USP21, Ubiquitin C-Terminal Hydrolase L3 (UCHL3), etc. in mammals (Artavanis-Tsakonas et al., 2006). Among these enzymes, CSN and NEDP1 are NEDD8-specific deneddylation enzymes. CSN is a metal zinc-containing protease containing eight subunits CSN1-8 (Rabut and Peter, 2008). Among them, CSN5 is the only CSN subunit with catalytic deneddylation activity, and it can only function in the context of the three-dimensional structure of the CSN full complex. The CSN complex inhibits the E3 ligase activity of CRL by removing NEDD8 from the Cullin adapter core (Chiba and Tanaka, 2004). CSN primarily removes NEDD8 from Cullin family proteins, whereas NEDP1 appears to be more specific for dissociating NEDD8 from Cullin family proteins (Mendoza et al., 2003). Studies have shown that NEDP1 can also remove NEDD8 from Cullin family proteins and non-Cullin family proteins in cell-free systems (Wu et al., 2003). CSN5-mediated deneddylation of Cullin family proteins is closely related to reducing microglial inflammation, attenuating brain endothelial inflammation, improving barrier integrity, and preventing ischemic stress-induced neuronal cell death. In addition, the researchers discovered 607 neddylation sites regulated by MLN4924 and NEDP1 by mapping the NEDP1 substrate repertoire. The 112th lysine residue of the actin regulatory protein Cofilin was selected as the research object, which has a great influence on the growth of neurons during brain development (Tian et al., 2023). Subsequent experimental results showed that global inhibition of actin neddylation resulted in cytoskeletal defects, altered actin dynamics, and impaired neurite outgrowth, indicating that the deneddylation process is critical in the establishment of the nervous system (Balachandran et al., 2016; Table 1).

In summary, the process of protein neddylation plays an important role in the nervous system by regulating multiple pathways, including regulating cell cycle progression, cell division, cell proliferation, signal transduction, DNA repair, participation in neural development, and neuronal apoptosis, death and autophagy, etc. Therefore, abnormal protein mimicry modification processes are associated with a variety of central nervous system diseases.

## 3 Protein neddylation participate various neurology diseases

### 3.1 Cerebrovascular disease

**Figure 2:**
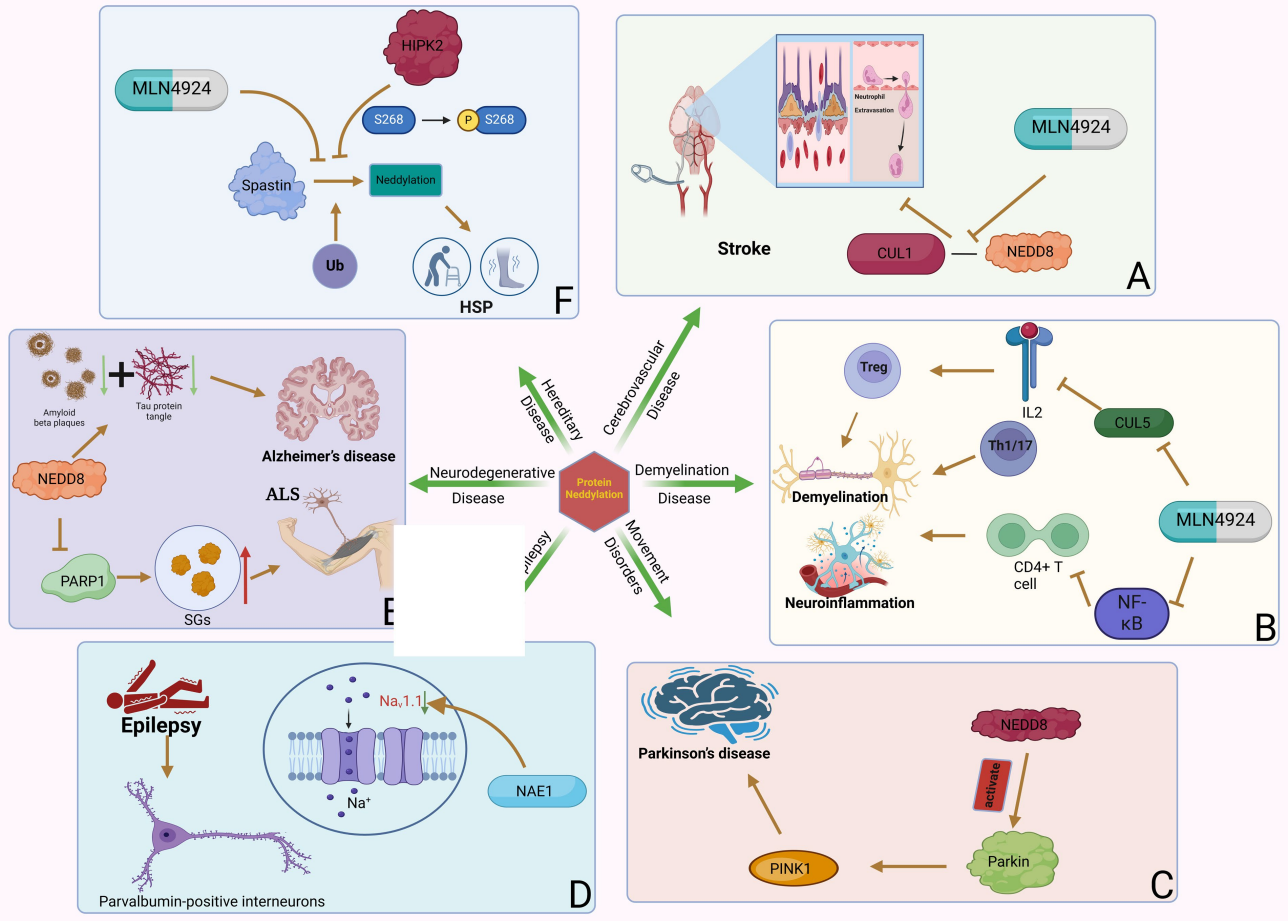
Neddylation can participate in various neurology diseases. Protein neddylation can promote the occurrence and progression of several central nervous system diseases through different mechanisms, while inhibitors of neddylation can reverse the development of the diseases. (A) NAE-mediated CUL1 neddylation in stroke increases neutrophil exosmosis and blood–brain barrier destruction, while MLN4924 can inhibit this process. (B) Neddylation of T cells induces neuroinflammation and demyelination in MS. (C) Neddylation of Parkin and PINK1 are important factors in the development of PD. (D) NAE1 deficiency reduces the excitability of PVIN by decreasing Nav1.1 channel activity, which can lead to epilepsy. (E) NEDD8-mediated neddylation promotes the development of ALS and AD by decreasing PARP1 activity and increasing the degradation of Aβ and tau proteins. (F) Neddylation of Spastin leads to the occurrence of HSP by promoting its degradation, and the phosphorylation of MLN4924 and HIPK2 can inhibit this process.

Cerebrovascular disease refers to brain dysfunction caused by cerebrovascular lesions or blood flow disorders caused by various reasons, including neurological dysfunction caused by vascular lumen occlusion, blood vessel rupture, blood vessel wall damage, or abnormal blood components. Stroke is an acute cerebrovascular disease that can be divided into hemorrhagic stroke and ischemic stroke. Stroke is the second leading cause of death and the third leading cause of disability worldwide (Hankey, 2017). An estimated 6.6 million Americans over the age of 20 suffer from a stroke, which occurs on average every 40 s and kills one patient every 4 min (Collaborators GBDLROS et al., 2018). In China, the stroke mortality rate is as high as 149.49/100,000 people, accounting for 22.33% of the total deaths (Wang et al., 2020). Early reports show that focal transient cerebral ischemia can induce neurogenesis in the adult brain. The upregulation of APPBP1 expression in neural progenitor cells after focal transient ischemia suggests that this protein contributes to neurogenesis induced by transient ischemia and reperfusion. One study found that 7 days after reperfusion in rats with focal transient cerebral ischemia, APPBP1 levels increased in the dentate gyrus and subventricular zone ipsilateral to the injury due to middle cerebral artery occlusion (MCAO; Joo et al., 2011). This suggests that APPBP1 plays a role in neurogenesis triggered by focal transient cerebral ischemia. A recent study found that the neddylation process was significantly upregulated in the post-stroke peri-infarct cortex following brief focal ischemia in mice (Yu et al., 2022). Inhibition of neddylation by the NAE inhibitor MLN4924 improves stroke prognosis by reducing neutrophil infiltration, attenuating blood–brain barrier damage and infarct volume, and improving neurological function. MLN4924 reduces neutrophil extravasation and blood–brain barrier disruption by weakening the binding of NEDD8 to CUL1 (Yu et al., 2022; Figure 2). A new study showed that MLN4924 not only reduced microglial inflammation and changes microglial morphology, but also reduced endothelial barrier leakage and neuronal death after ischemic stroke (Zhang, 2019). Therefore, MLN4924 may be a treatment modality for ischemic stroke.

### 3.2 Demyelination disease of CNS

Multiple sclerosis (MS) is an autoimmune disease characterized by inflammatory demyelination of the white matter of the central nervous system as its main pathological feature (Lin et al., 2023). While both genetic and environmental factors contribute to the onset of MS, its cause remains elusive. The pathogenesis of MS presents an autoimmune mechanism in which the target is represented by myelin antigens. Both CD4^+^ and CD8^+^ T cells play a role in pathological mechanisms (Hedstrom et al., 2021). In MS, pathogenic Th1, Th17, and autoreactive CD8+ T cells generate immune responses against myelin components. Furthermore, in demyelinating lesions, local microglia and macrophages are also activated (Gresle et al., 2020). In addition, B cells also play a role in the immunopathogenesis of MS. In the early stages of the disease, CD20^+^ B cells are most common, while in the advanced stages, plasmablasts and plasma cells are mainly involved (Ramesh et al., 2020). The participation of B cells is not only related to the production of antibodies but also to the functional regulation of T cells and bone marrow cells. Some treatments that antagonize the effects of immune cells can be effective in some patients (Hauser et al., 2008).

A transcriptomic study showed that neddylation-related genes were up-regulated in T cells of MS patients. In particular, NAE1 is significantly upregulated in CD4^+^ T cells. Some E3 subunits related to NAE1, such as Ankyrin Repeat and SOCS Box Containing 7, Leucine Rich Repeat Containing 41, WD and Tetratricopeptide Repeats 1 and F-box and Leucine Rich Repeat Protein 22, are related to the neddylation process in MS. These E3 subunits may play an important role in affecting CD4^+^ T cell function in MS (Kim et al., 2021). Experimental autoimmune encephalomyelitis (EAE) is a mouse model of MS driven by an established CD4^+^ T cell response. Researchers using MLN4924 in EAE found that MLN4924 significantly reduced the severity of EAE compared with the placebo treatment group (Robinson et al., 2014). This may be through inflammation of the nervous system and additional protection of axonal integrity. The difference in disease severity was specifically reflected in the stable body weight, significantly reduced demyelination, and significantly reduced spinal cord inflammation (especially in Iba1+ myeloid cells and CD3+ T cells, accompanied by a significant decrease in axonal loss) during the course of the disease in animals treated with MLN4924 compared with the placebo group. Mechanistically, inhibiting neddylation in CD4^+^ T cells can inhibit T cell proliferation and cytokine production by inhibiting the NF-κB pathway and increasing SOCS1 and SOCS3 expression, thereby inhibiting T cell function (Cheng et al., 2018). The NF-κB pathway is important in the pro-inflammatory response of immune cells. The neddylation pathway degrades the neddylation of NF-κB inhibitor and TNF receptor associated factor 6 through the ubiquitin-proteasome system (UPS), thereby activating the NF-κB pathway (Liu et al., 2019). Therefore, inhibiting neddylation also inhibits the activation of the NF-κB pathway, thereby reducing neuroinflammation and axonal damage (Figure 2).

Furthermore, not only does NAE1 have a strong functional effect on pathogenic T cell proliferation in MS, but MLN4924 also has potential pleiotropic effects on other immune subsets, including B cells and myeloid cells, but not astrocytes (Chang et al., 2012). Defects in IL-2 receptor (IL-2R) signaling in regulatory T cells (Treg) lead to weakened Treg function and are the basis of autoimmune diseases such as MS. GRAIL, a ubiquitin E3 ligase, is important for Treg function. GRAIL exerts a negative regulatory effect on IL-2R desensitization by ubiquitinating a lysine on CUL5 that must undergo neddylation to allow Cullin ring ligase activation. Neddylation inhibitors combined with low-dose IL-2 activators can be used to replace GRAIL and restore Treg function and stability in MS patients (Fathman et al., 2022). Therefore, neddylation may be a new therapeutic target in multiple sclerosis and can be used in combination with other disease-modifying drugs.

### 3.3 Movement disorders

Movement disorders, also known as extrapyramidal diseases, are a group of neurological diseases characterized by motor symptoms such as slow voluntary movements, involuntary movements, abnormal muscle tone, and posture and gait disorders. Parkinson’s disease (PD) is a degenerative neurological disease common in middle-aged and older people, clinically characterized by resting tremor, bradykinesia, muscle rigidity, and postural balance disorder (Blesa et al., 2023). The pathological hallmark of Parkinson’s disease is the progressive degeneration and death of dopaminergic neurons in the substantia nigra complex (Choo et al., 2012). In Parkinson’s disease, specific immunoreactivity to NEDD8 was detected in Lewy bodies, suggesting that protein neddylation is involved in the pathogenesis of Parkinson’s disease (Mori et al., 2005).

Parkin and PINK1 mutations are the main causes of autosomal recessive inheritance and early-onset Parkinson’s disease, with frequent Parkin mutations accounting for 77% of juvenile Parkinson’s disease and 10–20% of Parkinson’s disease in young patients (Wang et al., 2006). Parkin is a RING/HECK hybrid ubiquitin E3 ligase with a ubiquitin-like domain at the N-terminus and a RING-IBR-RING domain at the C-terminus (Wenzel et al., 2011). It mediates the ubiquitination and degradation of multiple proteins. In addition, Parkin plays a vital role in protecting neurons from various injuries and maintaining mitochondrial integrity (Deng et al., 2008). PINK1 is a putative kinase with an N-terminal mitochondrial targeting signal (Park et al., 2006). Although PINK1-mediated protein phosphorylation is unclear, several potential substrates of PINK1 have been identified (Murata et al., 2011). Parkin and PINK1 can undergo neddylation regulation. Neddylation modification leads to increased ubiquitin E3 ligase activity of Parkin and stabilization of the 55 kDa PINK1 proteolytic fragment (Choo et al., 2012). NEDD8 can upregulate ubiquitination levels and promote the degradation of a variety of misfolded proteins by coupling and activating Parkin E3 ligase. Ubiquitination of Parkin and synphilin-1, another substrate of E3 ligase, was also upregulated with increased neddylation in a dose-dependent manner. This indicates that NEDD8 enhances the activity of Parkin E3 ligase (Um et al., 2012; Figure 2). *In vivo*, enhanced neddylation by dAPP-BP1 overexpression rescued aberrant phenotypes induced by dPINK1 reduction in two independent dPINK1-RNAi Drosophila lines. This suggests that neddylation can rescue the reduction of PINK1 and stabilize the hydrolyzed fragments of PINK1, thereby inducing intracellular aggregation of PINK1. In addition, Choo et al. found through Western blot analysis that NEDD8 binding in the Parkinson’s disease pathological marker LB specifically modified Parkin and PINK1 (Ilic et al., 2022). The E3 ligase complex formed by Parkin, PINK1, and DJ-1 can promote the degradation of misfolded or unfolded proteins. The PD-related neurotoxin 1-methyl4-phenylpyridinium (MPP^+^) can also inhibit the neddylation of Parkin and PINK1. These findings indicate that the modification of Parkin and PINK1 by NEDD8 is involved in the pathogenesis of PD. The study also opens up a new avenue for potential PD treatment by modulating the neddylation of Parkin and PINK1.

### 3.4 Epilepsy

Epilepsy is a chronic neurological disorder characterized by recurrent seizures (Sinha et al., 2023). Epilepsy is a brain disorder whose main characteristic is a tendency to have ongoing seizures. These abnormal episodes of hypersynchronous network activity may result from a variety of brain insults, including developmental defects, acquired pathologies (trauma, stroke, infection), and myriad associated genetic defects. Mutations that disrupt ion channel genes, so-called channelopathies, are often found in familial epilepsy syndromes or identified as *de novo* lesions in developmental epileptic encephalopathies (Cannon, 2021). It affects people of all ages and has social, behavioral, health and economic consequences for patients and their families. It is estimated that more than 50 million people worldwide are affected by epilepsy. In the central nervous system, appropriate neural activity between excitatory pyramidal neurons (PyN) and inhibitory interneurons is critical for brain function. Parvalbumin-positive interneurons (PVINs) are necessary to generate local circuit oscillations and connections or synchronization between brain regions (Donato et al., 2013). The excitability of PVINs requires the voltage-gated sodium channel α subunit Nav1.1, which is specifically expressed in PVINs (Wang et al., 2011). Mutations in the Nav1.1 channel have been found in patients with both mild and severe epilepsy (Claes et al., 2001). The unified loss-of-function hypothesis proposes that mild impairment of Nav1.1 function results in febrile seizures, moderate loss of function results in generalized epilepsy with febrile seizures plus epilepsy, and severe functional impairment results in refractory epilepsy and severe myoclonic epilepsy of infancy comorbidities (Catterall et al., 2010).

Recently, researchers found that neddylation is related to the development of neuromuscular junctions and excitatory synapses on PyN (Vogl et al., 2015). Many neddylation substrates do exist in PVIN, and the downstream effects of this post-translational modification are likely to be diverse and widespread. NAE1 is an essential subunit of the identified NEDD8 E1 in PVIN. Mice with mutations in NAE1 develop ataxia, spontaneous seizures, impaired inhibitory synaptic transmission, and reduced excitability (Figure 2). Specifically, NAE1 mutant mice initially exhibited limb tremors, unbalanced or uncoordinated walking, or stumbling. Subsequently, the mutant mice developed an ataxia-like phenotype compared with control mice in a balance beam test. The mutant mice exhibited seizures over time, and the frequency and severity of seizures increased with age. Eventually, the mutant mice began to die, either due to seizures or sudden and unexpected death. These results demonstrate the critical role of PVIN’s NAE1 in regulating brain activity. However, morphological studies have shown that this mutation has no significant effect on the number of PVINs in the cortex and hippocampus (Chen et al., 2021).

Notably, reduced GABA release in mutant mice subsequently leads to increased PyN firing and glutamate transmission, which correlates with reduced PVIN excitability, suggesting that neddylation is critical for the control of inhibitory interneuron excitability. Further molecular mechanism studies determined that NAE1 deficiency reduces Nav1.1, thereby reducing PVIN excitability, ultimately leading to epilepsy. Restoring Nav1.1 in neddylation-deficient PVIN treated with MLN4924 is sufficient to restore excitability, indicating that Nav1.1 deficiency is the primary mechanism by which NAE1 mutations regulate PVIN excitability. And Nav1.1 requires neddylation to be stably expressed in PVIN. This was also supported by proteomic analysis, which also revealed abnormalities in synaptic and metabolic pathways. Finally, rescue expression of Nav1.1 in mutant mice attenuated the increase in PVIN excitability. In summary, insufficient neddylation in PVIN reduces the stability of Nav1.1, thereby reducing the excitability of PVIN; the subsequent increased PyN activity causes seizures in mice. Conversely, defects in neddylation reduce the stability of Nav1.1 in PVIN, thereby reducing epileptic seizures (Chen et al., 2021). Further studies are needed to explore whether modification of neddylation represents a new therapeutic strategy for epilepsy.

### 3.5 Neurodegenerative disease

Neurodegenerative diseases are a group of chronic and progressive diseases of unknown origin that damage tissues such as the central nervous system and peripheral nervous system. Amyotrophic lateral sclerosis (ALS) is a neurodegenerative disease that affects the locomotor system and leads to the degeneration of motor neurons in the brain’s motor cortex, brainstem and spinal cord (Liu et al., 2023). The resulting disruption in communication between the nervous system and skeletal muscles is the characteristic pattern that initially leads to muscle weakness and spasticity. As muscle denervation progresses, patients develop paralysis, and most patients will die of respiratory failure within 3–5 years of diagnosis. In ALS, protein mutations found in cytoplasmic condensates called stress granules (SGs) are associated with pathological SG formation, abnormal protein inclusions, and neuronal toxicity. One study found that inhibition of NEDP1 promoted physiological and pathological SG breakdown (Kassouf et al., 2023). Reduced activity of poly ADP-ribose polymerase 1 (PARP1) through NEDD8 neddylation is a key mechanism for the observed phenotype. Crucially, inhibition of NEDP1 also promoted the breakdown of abnormal SGs in ALS patient-derived fibroblasts. In *Caenorhabditis elegans*, deletion of NEDP1 improves ALS phenotypes related to animal movement. Based on the above-mentioned role of PARP-1 in SG dynamics, PARP1 inhibitors have been proposed as an attractive therapeutic approach to eliminate abnormal SGs (Grimaldi et al., 2019). Targeting NEDP1 can provide the desired PARP-1 regulation, reduce PARP-1 overactivation, and promote the breakdown of pathogenic SGs without any toxic effects. This study reveals NEDP1 as a potential target for ameliorating ALS-related phenotypes and provides justification for the use of anti-NEDP1-Nb as an attractive ALS therapeutic.

Alzheimer’s disease (AD) is a more common degenerative disorder of the central nervous system characterized by progressive cognitive impairment and behavioral impairment. Two important pathological features of AD are the abnormal deposition of amyloid Aβ and the hyperphosphorylation of tau to form neurofibrillary tangles, accompanied by massive neuronal apoptosis and neuroinflammation (Yamazaki et al., 2019). The accumulation of Aβ can lead to a series of downstream effects, such as neurofibrillary tangles, inflammation, excitotoxicity, oxidative stress, etc. The accumulation of insoluble proteins in the AD brain may be caused by UPS overload or dysfunction or by conformational changes in protein substrates that prevent UPS degradation and recognition. Neddylation can modify the E3 ligase of UPS to promote the degradation of Aβ and hyperphosphorylated tau (Al Mamun et al., 2020; Figure 2). APP is the precursor of Aβ and can undergo neddylation on every lysine residue in its intracellular domain, which is thought to regulate APP processing and signaling (Soucy et al., 2010). APP induces neuronal apoptosis by binding to APP-BP1, which is the regulatory subunit of the NEDD8 activating enzyme (Chow et al., 1996). Increased APPBP1 in lipid rafts was found in AD-affected hippocampus, and NEDD8 was transported from the nucleus to the cytoplasm in AD hippocampal neurons (Balasubramaniam et al., 2019). Neddylation of Cullin-1 is necessary for the function of SCFβTrCP, linking the ubiquitin and NEDD8 pathways in the regulation of targeted protein degradation. The neddylation process also affects the cell cycle by inhibiting the G1-S transition via CUL1-based E3 ligase (Read et al., 2000).

Studies also show that elevated IL-1 levels in neuroinflammation mediate the translocation of NEDD8 from the nucleus to the cytoplasm, where it binds to the ubiquitin ligase E3 parkin to activate subsequent degradation of misfolded proteins. This process is neuroprotective. NEDD8 is involved in cell cycle regulation, apoptosis, cell death and other life processes in the nucleus (Kim et al., 2021). Translocation of NEDD8 to the cytoplasm by IL-1 corresponds to the downregulation of neddylation expression in the nucleus. Therefore, it is speculated that IL-1 may promote the degradation of misfolded proteins by modifying CUL-1 through NEDD8. Another study showed that neddylation regulates Aβ metabolism. Presenilin 1 is a component of γ-secretase, which acts on APP and produces Aβ. Presenilin 1 can be ubiquitinated by CRL1, and the activation of CRL1 depends on the neddylation regulation of Cullin-1. APP-BP1 promotes the ubiquitination of PS1 and reduces the production of Aβ (Li et al., 2002). Furthermore, APP-BP1 activates parkin-containing E3 and promotes its degradation via Lys-63-linked ubiquitin chains (Olzmann et al., 2007).

It was previously reported that NUB1/NUB1L interacts with accumulated hyperphosphorylated tau protein and significantly reduces levels, but NUB1 has an inhibitory effect on the proteasome. This suggests that NUB1 reduces tau aggregation in cells through other pathways (Richet et al., 2012). Guarascio et al. found that proteasome damage led to a significant increase in p62 levels, and NUB1 regulated autophagosome function and induced the accumulation of lysosomes. This suggests that NUB1 may clear the accumulation of highly phosphorylated tau protein by regulating the autophagy-lysosomal pathway (Guarascio et al., 2020). Furthermore, published data suggest that Aβ pathology is very similar to the “Western diet” promoting inflammatory processes in the brain (Wieckowska-Gacek et al., 2021). Aβ pathology and insulin resistance have functional interactions in AD (Chatterjee and Mudher, 2018) and can lead to the worsening of AD symptoms. One study shows that inhibition of neddylation of Cullins at synaptic sites preserves synaptic insulin signaling and rescues memory deficits and synaptic plasticity in mice with high amyloid load fed a “Western diet.”

### 3.6 Hereditary disease of CNS

Hereditary spastic paraplegia (HSP) is a multigene monogenic neurological disorder that causes corticospinal and dorsal axonal atrophy of the spinal cord, with an incidence of 0.1–9.6, affecting 1 in 100,000 people worldwide. Key manifestations of HSP include bilateral spasticity of the lower extremities, hyperreflexia, plantar extensor reflexes, muscle weakness, and induced gait deviations (Chen et al., 2023). HSP is caused by autosomal dominant mutations in the SPG4 gene encoding the microtubule-cleaving protein spastin. Spastin is an ATPase microtubule severing enzyme that regulates cytoskeletal rearrangements associated with membrane remodeling. It is a key regulator of cell division and nuclear membrane resealing during cell division and is also involved in intracellular trafficking (Allison et al., 2013). Spastin is highly expressed in the nervous system (Schmitz et al., 2014) and plays a key role in axonal transport and regeneration (Stone et al., 2012). Increasing the levels of the wild-type spastin allele may be an effective treatment for HSP.

Recently, researchers discovered that spastin is a new target of the multifunctional kinase HIPK2 (Pisciottani et al., 2019) and is post-transcriptionally regulated by HIPK2 protein levels in proliferating cells, differentiated neurons, and *in vivo*. Further analysis found that HIPK2-mediated phosphorylation of S268 contributes to the stability of spastin and protects spastin from K554-mediated polyubiquitination and degradation (Sardina et al., 2020). Ubiquitination experiments were performed using the K48-only Ub mutants model, and it found that this mutant only allows K48-linked polyubiquitination chains, which is a typical proteasomal degradation signal. Testing the K554 mutant of Spastin for ubiquitination revealed that spastin is polyubiquitinated at K554R. In addition, Cullin-Associated NEDD8-Dissociated Protein 1 (CAND1) is a known inhibitor of the CRL complex, and its interaction with spastin suggests that the neddylation pathway is involved in the degradation of spastin through UPP (Soucy et al., 2009). In conclusion, HIPK2-mediated phosphorylation of spastin at S268 inhibits the multiple ubiquitination of spastin K48 at K554 and prevents its neddylation-dependent proteasomal degradation (Figure 2). In a spastin RNAi neuronal cell model, overexpression of HIPK2 or treatment with a low, non-toxic dose of MLN4924 restored spastin levels and rescued neurite defects. In spastin mouse model-derived neurons and patient-derived cells of HSP, spastin levels can be pharmacologically restored by inhibiting its neddylation-mediated degradation, revealing a novel therapeutic target for SPG4-HSP treatment.

## 4 The dual role of protein neddylation in glioma and targeted therapeutics

### 4.1 Neddylation can promote the proliferation of GBM cells

Primary brain tumors refer to a heterogeneous group of tumors arising from cells within the central nervous system. Among them, neuroepithelial tumors are the most common primary brain tumors in adults (Ostrom et al., 2017). Gliomas are neuroepithelial tumors that account for more than 70% of adult malignant brain tumors, among which Glioblastoma multiforme (GBM), which is World Health Organization (WHO) grade IV, is the most serious. The latest statistics show that the incidence rate of GBM is 3.2/100,000 people (Ostrom et al., 2019). According to the current treatment plan of maximum surgical resection combined with postoperative standard radiotherapy or chemotherapy, the median survival time of patients is less than 2 years. The alkylating agent Temozolomide (TMZ) is currently one of the most widely used chemotherapy drugs. However, TMZ resistance has always been one of the important reasons affecting the efficacy of chemotherapy (Stupp et al., 2015). GBM is an invasive, highly drug-resistant, and ultimately incurable disease that requires continued research into its inherent biology to identify novel therapeutic avenues. In recent years, researchers have also continued to work on finding new effective targets for the treatment of GBM. Since the protein neddylation process is involved in DNA damage repair, epigenetic changes, cellular drug efflux and other related biological processes, targeting neddylation to treat GBM may have good results (Singh et al., 2021).

**Figure 3:**
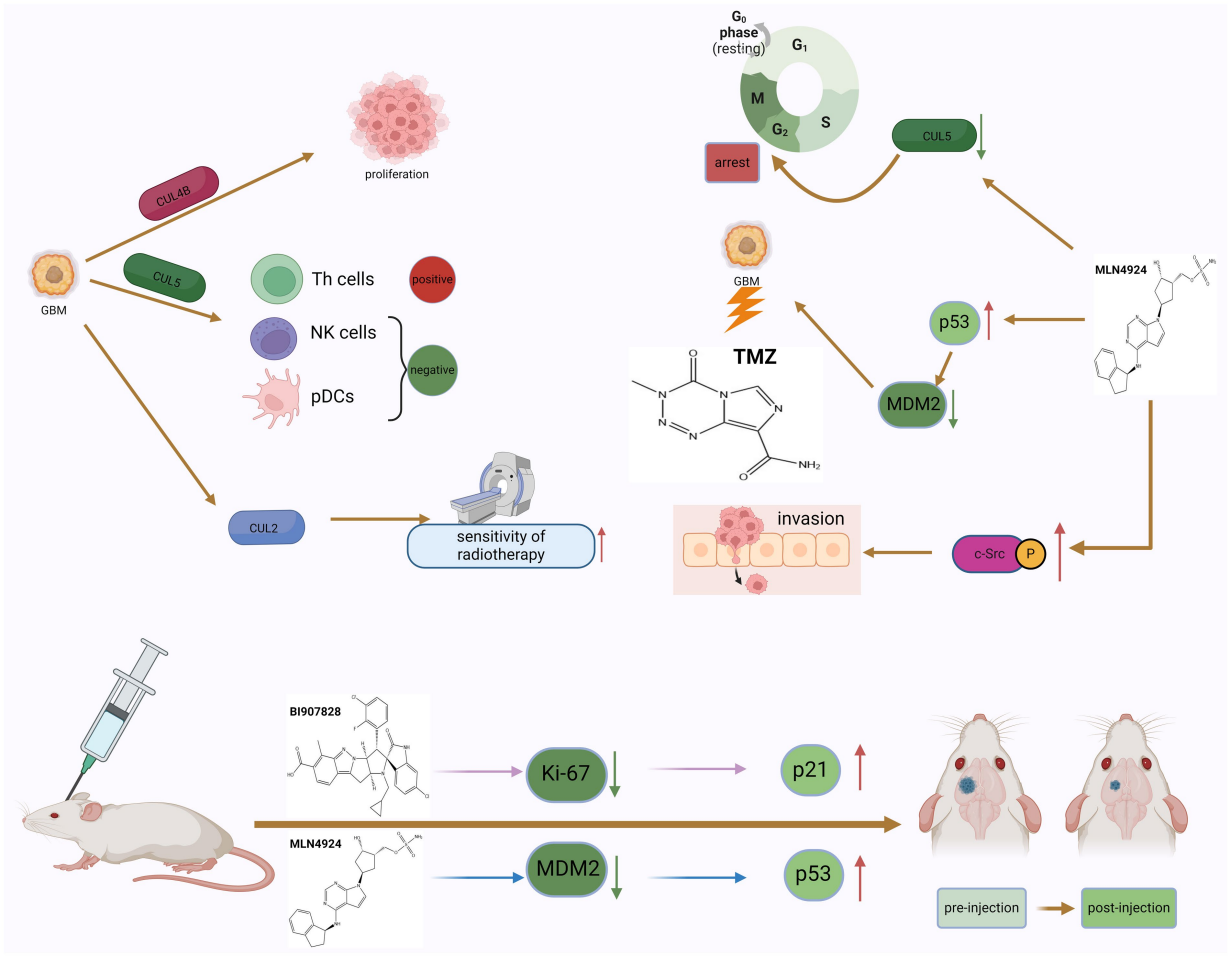
The dual role of neddylation in GBM and the potential therapeutic effects of inhibitors. The phenomenon of neddylation in GBM can promote the proliferation of GBM cells and the infiltration of immune cells, but it also increases the sensitivity of GBM to radiotherapy. The use of MLN4924 to inhibit the mimetic process can induce GBM cells to remain in the G2 phase of the cell cycle and increase the killing effect of TMZ, but it also promotes the invasion of GBM cells. The use of neddylation inhibitors in tumor bearing mice can improve their survival rate, indicating that targeted neddylation therapy is a potential therapeutic approach for GBM.

CUL4B has been shown to be significantly upregulated in various types of solid tumors and has potent oncogenic properties (Liu et al., 2012). MicoRNA-674-5p can bind to the 3’-UTR of CUL4B mRNA and inhibit the expression level of CUL4B. In GBM, micoRNA-674-5p can bind to the 3’-UTR of CUL4B mRNA and inhibit the expression level of CUL4B, thereby inhibiting the proliferation and migration of GBM cells (Li et al., 2022). Furthermore, CUL5 is one of one of the most typical neddylation substrates. The researchers found that there were differences in the expression levels of CUL5 in GBM tissues and adjacent normal tissues. Studies have shown that the expression of CUL5 may regulate tumor prognosis by changing and regulating the infiltration of certain immune cells. CUL5 is positively correlated with Th cells and negatively correlated with pDCs and NK cells (Li et al., 2021). Elevated expression of RNF7, TCEB1, SOCS1, and SOCS3 genes encoding CRL5 components can predict poor prognosis of GBM, and strategies targeting CRL5 may inhibit the progression of GBM (Figure 3).

### 4.2 Inhibitors of neddylation process can exhibit anti-tumor activity

MLN4924 is an adenosine 5′-monophosphate analog that binds to the nucleotide-binding site of NEDD8 activating enzyme (Soucy et al., 2009). Many studies have reported that MLN4924 exhibits cytotoxic effects against cancer cells and xenografted tumors and inhibits tumor progression (Luo et al., 2012). MLN4924 can inhibit the neddylation of CUL and lead to the accumulation of CRL substrates, ultimately causing DNA damage, cell cycle arrest and apoptosis in tumor cells. Based on preclinical studies, MLN4924 has potent anti-tumor activity against solid tumors and hematological malignancies. In addition, in phase I/II clinical trials, MLN4924 showed promising clinical efficacy and low toxicity. The combination of MLN4924 with chemotherapeutic drugs improved antitumor activity in solid tumor cell lines and xenograft models (Zhou et al., 2018). MLN4924’s ability to penetrate the blood–brain barrier suggests that the drug may play a role in treating GBM. Hua et al. systematically studied the effectiveness of MLN4924 against GBM *in vitro* and *in vivo*. Trial results show that patients with higher levels of neddylation have lower survival rates, and inhibition of the neddylation process induced by MLN4924 can improve the survival rate of patients (Hua et al., 2015). The researchers also found that GBM expressed higher levels of NEDD8 and related enzymes, such as NAE1, UBA3, and UBE2M, compared with normal brain tissue surrounding the tumor. In addition, the degree of protein neddylation is more severe in recurrent GBM. MLN4924 can induce GBM cells to arrest in the G2 phase of the cell cycle, and subsequently induce senescence and apoptosis of GBM cells. On the other hand, experimental results in mice also show that MLN4924 can inhibit tumor growth. Immunohistochemical analysis found that in tumor sections of MLN4924-treated xenograft mice, the levels of the cell proliferation marker Ki-67 were reduced, while the expression of the cell cycle inhibitor p21 was increased (Figure 3). These findings further support that MLN4924 can be considered an effective treatment modality for GBM (Mansouri and Zadeh, 2015).

MLN4924 can also increase the sensitivity of GBM cells to TMZ (Figure 3). Studies have shown that the combined application of MLN4924 and TMZ can effectively reduce the viability of highly TMZ-resistant GBM cells and induce cell apoptosis. Interestingly, MGMT protein expression was reduced in cells treated with MLN4924 compared with TMZ treatment alone, suggesting that this drug combination interferes with MGMT expression or degradation to some extent (Polivka et al., 2017). MGMT has an inverse relationship with the expression of tumor protein 53 (p53) in GBM cells treated with MLN4924. P53 is a key tumor suppressor protein. As a transcription factor, p53 regulates multiple downstream target genes that are involved in cell cycle arrest or senescence, DNA repair, and apoptosis (Hu et al., 2023). In cells that are exposed to stress signals or are damaged, p53 is rapidly activated while it is kept in check in normal cells. When the p53 gene is mutated or the p53 protein function is inhibited, it will cause the proliferation of a variety of tumor cells (Lawrence et al., 2014). Studies have confirmed that deletion and mutation of p53 gene and inactivation of p53 protein promote the occurrence and development of GBM. At the same time, restoring the function of p53 protein helps inhibit the growth of GBM (Mitobe et al., 2022). As an upstream molecule of p53, MDM2’s amplification and overexpression can induce the inactivation of p53 and downstream tumor suppressor effect substances, thereby promoting the proliferation of glioblastoma cells. In human GBM cells, MGMT expression is affected by down-regulation of p53 (Bocangel et al., 2009), and neddylation modification of p53 can inhibit its transcriptional activity (Xirodimas et al., 2004). Therefore, inhibition of the overactivated neddylation pathway in GBM by MLN4924 may increase the transcriptional activity of p53, thereby downregulating MGMT. This effect was not evident in single MLN4924 treatment but was evident in combination treatment. The results of another trial on the combination of TMZ and MLN4924 in the treatment of GBM also showed that the combination of TMZ and MLN4924 can inhibit MGMT-mediated DNA repair by enhancing p53-mediated MGMT inhibition and may be responsible for the increased sensitivity of GBM to TMZ (Brandt et al., 2023). In addition, Filippova et al. also evaluated the effect of MLN4924 on glioma PD-L1 expression and glioma cell immune evasion. T cell-based analysis confirmed that upregulation of PD-L1 in tumor cells protected GBM from T cell attack and reduced T cell activation. After MLN4924 treatment, HIF1A and PD-L1 mRNA and protein levels were significantly increased in all glioma cell lines. This study demonstrates that PD-L1 upregulation in gliomas and the glioma microenvironment is an important target; MLN4924, combined with blockade of the PD1/PD-L1 pathway, should be considered a potential candidate for glioma treatment strategy (Filippova et al., 2018).

BI-907828 is an MDM2-specific inhibitor that blocks the interaction between MDM2 and p53 by binding to MDM2. This prevents MDM2 from inactivating p53, thereby restoring p53 function in tumor cells and inducing the expression of target genes involved in processes such as cell cycle arrest and DNA repair, senescence, and apoptosis (Cornillie et al., 2020). *In vitro* cell experiments have shown that BI-907828 can specifically inhibit the expression of MDM2 and restore the expression levels and activities of tumor suppressor factors such as p53 and p21, thereby reducing the viability of GBM cells and inducing apoptosis (Figure 3). Experiments in mice further showed that BI-907828 combined with TMZ treatment can improve the survival rate of mice (Hao et al., 2023).

### 4.3 The protective activity of neddylation process in GBM

Since neddylation is essential for rapid protein turnover in cancer cell proliferation, it is currently considered a potential target for cancer therapy (Olaizola et al., 2022). However, in one study, researchers found that MLN4924 promoted the formation of tumor spheroids and stem cell differentiation, and stimulated the spread and metastasis of cancer cells (Zhou et al., 2016). Both MLN4924 and NEDD8 knockdown stimulated GBM cell migration by inducing the expression of c-Src and the phosphorylation of c-Src at tyrosine 416. Researchers found that C-CBL is an E3 ligase for c-Src neddylation, and neddylated c-Src undergoes ubiquitination and proteasomal degradation. After neddylation, c-Src is poly-ubiquitinated and degraded through the proteasome, which inhibits the PI3K/AKT pathway responsible for cell migration. Therefore, C-CBL is likely to play a tumor suppressive role by antagonizing a robust oncogenic signaling driven by c-Src. C-CBL expression is negatively correlated with c-Src/AKT phosphorylation, GBM metastasis, and patient survival (Figure 3).

Therefore, C-CBL is likely to play a tumor suppressive role by antagonizing a robust oncogenic signaling driven by c-Src. This suggests that neddylation may be an important mechanism in inhibiting GBM invasion and spread (Lee et al., 2018). Treatment of GBM includes maximal surgical resection and radiation therapy based on the STUPP trial, and combined with TMZ chemotherapy. Despite intensive treatment, GBM eventually becomes resistant to RT and TMZ, and most patients will succumb to the disease, with a median survival of only 15 months (Lee et al., 2013). Studies have shown that tumor resistance to radiotherapy is closely related to the activation of epidermal growth factor receptor (EGFR), and the amplification of EGFR in GBM results in reduced sensitivity of GBM to radiotherapy (Eskilsson et al., 2018). Therefore, a new method to measure EGFR levels in GBM is urgently needed to classify GBM patients with different susceptibility to radiosensitive treatments. Studies identified that cullin2-RING E3 ligase (CRL2) mediates c-Cbl-independent ubiquitin conjugation and lysosome-independent degradation of the activated EGFR (Zhou and Yang, 2011). Besides EGFR, another major substrate of CRL2 is hypoxia-inducible factor 1α (HIF-1α) which is critically involved in GBM angiogenic activities. Degradation of both EGFR and HIF-1α requires von Hippel–Lindau (VHL) as a substrate recruiting protein in the CRL2 complex (CRL2VHL; Wang et al., 2016). Experimental results show that encoded in CRL2 Expression levels of the scaffolding protein CUL2 can predict GBM progression and survival. CUL2 protein levels were negatively correlated with levels of HIF-1α, VEGF-A, Cyclin B1 and EGFR. Increased expression of CUL2 predicts increased sensitivity of tumor cells to radiation (Zheng et al., 2021). Therefore, in the process of targeting neddylation to treat GBM, the dual effects of neddylation must be taken into consideration, and further mechanisms require more in-depth research to elucidate.

## 5 The role of protein neddylation in other CNS tumors

### 5.1 CRL4-DCAF11 mediated neddylation can promote the development of meningioma

Meningioma is the most common intracranial tumor that arises from the arachnoid and dural border cells of the leptomeninges surrounding the brain and spinal cord (Ostrom et al., 2015). The primary treatment for meningioma is surgery followed by radiotherapy (Wiemels et al., 2010). However, complete resection is not always possible, and recurrence is more likely, particularly in patients with higher grade meningioma (Gousias et al., 2016). Merlin is a tumor suppressor protein that inhibits tumor cell proliferation by inhibiting or activating relevant cell signaling pathways and preventing cytoskeletal re-combination (Morrison et al., 2007). Studies have found that the abnormal mutation of merlin in meningiomas, which can increase the activity of CRL4 as NEDD8 E3 ligase, may promote the binding of CRL4 and its specific substrate protein DDB1 And CUL4 Associated Factor 1 (DCAF1), induce the mimicinization of DCAF1, and then promote the proliferation of meningioma cells (Angers et al., 2006). In addition, as a scaffold protein in the Raf/MEK/ERK pathway, the activity of the kinase suppressor of Ras 1 (KSR1) was closely related to the activity of the CRL4-DCAF1 complex (Zhou et al., 2016). The researchers found significantly elevated levels of the CRL4-DCAF1 complex and KSR1 protein in meningioma cells compared to normal arachnoid cells. MLN3651 (Selumetinib), a neddylation specific inhibitor, can reduce the viability of meningioma cells, inhibit the proliferation of meningioma cells, and induce the apoptosis of meningioma cells. In addition, the researchers also confirmed that the use of MLN3651 or knocking down DCAF1 can lead to the loss of activity of KSR1 protein in meningioma cells, which in turn lead to the inactivation of the Raf/MEK/ERK pathway, inhibiting the proliferation of meningioma cells (Lyons Rimmer et al., 2020).

### 5.2 Targeting IAP-mediated p21 protein neddylation can inhibit MB cells proliferation

Medulloblastoma (MB), an embryonal tumor originating in the cerebellum, is the most common type of malignant primary brain tumor in children, accounting for 15–30% of all central nervous system tumors in children. The standard treatment for MB is maximum surgical resection combined with radiotherapy and chemotherapy; however, most patients still die from disease progression due to the tumor’s susceptibility to recurrence and metastasis (Dolecek et al., 2012). Inhibitors of apoptosis proteins (IAP) are highly expressed in MB cell lines and tissues, and previous studies have demonstrated that IAP specific inhibitors (LCL161 or LBW242) can activate caspase-3 and caspase-7 proteins in MB cells, and activate MB cells to apoptosis and autophagy (Chen et al., 2016). IAP is a highly conserved protein, and there are three kinds of IAP in human cells: X-linked IAP (XIAP), cellular IAP1 (cIAP1), and cellular IAP2 (cIAP2). They have conserved regions including baculovirus IAP repeats (BIR) and the RING domains (Kocab and Duckett, 2016). Interestingly, the RING structure of IAP can act as NEDD8 E3 Ligase, which can induce the neddylation of the downstream target proteins of IAP (Kamada, 2013). In MB cells, IAP can induce cyclin-dependent kinase inhibitor (CKI) p21 to undergo cytoplasmic modification, resulting in p21 being hydrolyzed by protease and losing its function of inhibiting cell cycle, while resulting in continuous proliferation of MB cells. IAP inhibitors can effectively block the p21 protein neddylation process in MB cells, resulting in the increase of active p21 content, and induce MB cells to stop in the G2/M phase of the cell cycle (Chen et al., 2018). The researchers confirmed that IAP inhibitors effectively reduced the expression of cyclin A, cyclin B1, cyclin-dependent kinase 1 (CDK1), and cyclin-dependent kinase 2 (CDK2), which in turn showed that targeting IAP-mediated p21 protein neddylation may become a new clinical strategy for MB treatment.

### 5.3 MLN4924 can regulate proteins associated with programmed cell death in DLBCL

Primary central nervous system lymphoma (PCNSL) refers to lymphoma confined to the central nervous system, accounting for 1–5% of primary intracranial tumors, the incidence of which is gradually increasing (Chen et al., 2018). 90% of PCNSL patients are diffuse large B-cell lymphoma (DLBCL), about half of which are multi-centric lesions that can occur in different parts of the brain at the same time. The main manifestations include headache, epilepsy, and focal motor dysfunction. At present, the diagnosis and treatment plan for PCNSL is a combination of surgical biopsy combined with radiotherapy and chemotherapy, among which methotrexate (MTX) is the first choice of first-line treatment for PCNSL (Radke et al., 2022). In DLBCL, the neddylation of CRL can induce the degradation of BCL-2 protein, which can promote apoptosis, leading to the imbalance of the apoptosis process and abnormal cell proliferation (Olejniczak et al., 2010). Studies have shown that MLN4924 can effectively inhibit NAE activity in DLBCL cells, resulting in a decrease in the level of CRL and an increase in the content of BCL-2 protein, and then induce the apoptosis of DLBCL cells (Torka et al., 2020). MLN4924 can also inhibit the growth of DLBCL cells by inhibiting primification, which triggered the arrest of DLBCL cells in the G2 cell cycle. Mln4924-mediated inhibition of neddylation can regulate the expression levels and activities of various programmed cell death related proteins in DLBCL cells. The study confirmed that MLN4924 can up-regulate the expression levels of pro-apoptotic proteins Noxa, Bil and p21 in DLBCL cells, while down-regulate the expression levels of anti-apoptotic proteins IAP in DLBCL cells (Wang et al., 2015). Meanwhile, MLN4924 can inhibit the NF-κB pathway activity in DLBCL cells, thereby inhibiting the proliferation of DLBCL cells (Godbersen et al., 2014).

## 6 Conclusions and future expectations

Neddylation is a post-translational modification that attaches NEDD8 to a substrate protein to affect its localization, stability, or activity. CRL can be activated through neddylation, thereby promoting ubiquitination and degradation of substrates, which then mediates many cellular processes, including cell cycle progression, apoptosis, and cell survival. In this review, we point out that neddylation may act through different mechanisms in different neurological diseases. For movement disorders, epilepsy, and neurodegenerative diseases, neddylation may have a protective effect against these diseases. Studies have shown that neddylation can increase the ubiquitin E3 ligase activity of the mutant gene parkin and stabilize the PINK1 proteolytic fragment. In addition, neddylation stabilized the expression of Nav1.1 in PVIN, thereby reducing epileptic seizures. In neurodegenerative diseases, inhibition of NEDP1 can promote the breakdown of abnormal SGs in ALS, providing justification for the use of anti-NEDP1-Nb as an attractive ALS therapeutic. For AD, neddylation seems to play a protective role in AD by modifying the E3 ligase of the UPS to promote the degradation of Aβ and hyperphosphorylated tau. On the contrary, for cerebrovascular diseases, demyelinating diseases, genetic diseases, and neurological tumors, excessive activation of neddylation may promote the development or progression of these diseases. MLN4924 is a first-in-class small molecule inhibitor of NEDD8 activating enzyme that selectively blocks CRL activation, thereby reducing proteasomal degradation of CRL substrates. MLN4924 significantly inhibits tumor cell growth by inducing senescence, autophagy and apoptosis, demonstrating preclinical efficacy in multiple cancer models. In cerebrovascular disease, inhibition of neddylation by MLN4924 improves stroke outcomes by reducing neutrophil extravasation, blood–brain barrier disruption, and neuronal death. In MS, inhibition of neddylation with MLN4924 reduces EAE disease severity. For HSPs, inhibition of neddylation restored spastin levels and rescued neurite defects. For nervous system tumors, the survival rate of GBM patients decreases with the degree of neddylation. MLN4924 inhibits the neddylation process in GBM by downregulating the activity of CRL5, inducing GBM cells to arrest in the G2 phase of the cell cycle, and subsequently inducing senescence and apoptosis of GBM cells to improve the survival rate of GBM patients. In addition, MLN4924 can also increase the sensitivity of GBM cells to TMZ. However, neddylation still has a certain protective effect on GBM. Neddylation can increase the sensitivity of GBM to radiotherapy and inhibit the spread and invasion of GBM. In this review, we point out that the neddylation inhibitor MLN4924 is a promising approach for the treatment of CNS diseases such as nervous system tumors and cerebrovascular diseases. However, the significance of neddylation in some other neurological diseases is unclear. Meanwhile, protein neddylation is also involved in the development and progression of meningioma, MB and PCNLS, while the use of specific inhibitors to inhibit the neddylation of protein substrates helps to inhibit the proliferation of tumor cells. In addition, there are still many gaps in neddylation activators and other neddylation inhibitors, and more research is urgently needed in the future to explore their effects on the nervous system to improve the prognosis of various central nervous system diseases.

## Glossary

Aβ: Amyloid Beta
AChR: Acetylcholine Receptor
AD: Alzheimer’s Disease
ALS: Amyotrophic Lateral Sclerosis
APP: Amyloid Precursor Protein
APPBP1: Amyloid Beta Precursor Protein-Binding Protein 1
ATP: Adenosine 5′-triphosphate
BIR: Baculovirus IAP Repeat
CBL: Cbl Proto-Oncogene
CDK1: Cyclin-Dependent Kinase 1
CDK2: Cyclin-Dependent kinase 2
cIAP1: cellular IAP1
cIAP2: cellular IAP2
CKI: Cyclin-dependent Kinase Inhibitor
CNS: Central Nervous System
CRL: Cullin-RING E3 Ubiquitin Ligase Complex
CSN: COP9 Signalosome
CUL: Cullin protein family
DCAF1: DDB1 And CUL4 Associated Factor 1
DCN1: Defective In Cullin Neddylation 1
DCUN1D1: Defective In Cullin Neddylation 1 Domain Containing 1
DCUN1D2: Defective In Cullin Neddylation 1 Domain Containing 2
DDB1: Damage Specific DNA Binding Protein 1
DLBCL: Diffuse Large B-cell Lymphoma
DLG4: Discs Large MAGUK Scaffold Protein 4
E1: NEDD8 E1 activating enzyme
E2: NEDD8 E2 conjugating enzyme
E3: NEDD8 E3 ligase
EAE: Experimental autoimmune encephalomyelitis
FBXO11: F-Box Protein 11
GBM: Glioblastoma multiforme
HSP: Hereditary Spastic Paraplegia
IAP: Inhibitor of Ppoptosis protein
IL: Interleukin
IL-2R: IL-2 receptor
KSR1: Kinase suppressor of Ras 1
MB: Medulloblastoma
MDM2: Murine Double Minute 2 Proto-Oncogene
MLN4924: Pevonedistat
MNAT1: Murine N-acetyltransferase 1
MPP^+^: 1-methyl-4-phenylpyridinium
MS: Multiple Sclerosis
MTX: Methotrexate
NAE1: NEDD8 Activating Enzyme E1 Subunit 1
NEDD8: Neural Precursor Cell Expressed Developmentally Downregulated Protein 8
NMJ: Neuromuscular Junction
p53: Tumor Protein 53
PARP1: Poly ADP-ribose Polymerase 1
PCNSL: Primary Central Nervous System Lymphoma
PD: Parkinson’s Disease
PF: Posterior Fossa
PS: Presenilin
PSD95: Postsynaptic Density Protein-95
PTM: Post-translational Modification
PVIN: Parvalbumin-positive Interneuron
PyN: Pyramidal Neuron
Rapsyn: Receptor-associated Protein at Synapse
RBX1: RING-box Protein 1
RBX2: RING-box Protein 2
RNF111: Ring Finger Protein 111
RNF168: Ring Finger Protein 168
SENP8: SUMO Peptidase Family Member, NEDD8 Specific
SG: Stress Granule
SP: Spinal Cord
ST: Supratentorial
TMZ: Temozolomide
Treg: Regulatory T cell
TRIM40: Tripartite Motif Containing 40
UBA3: Ubiquitin Like Modifier Activating Enzyme 3
UBE2F: Ubiquitin Conjugating Enzyme E2 F
UBE2M: Ubiquitin Conjugating Enzyme E2 M
UCHL3: Ubiquitin C-Terminal Hydrolase L3
USP21: Ubiquitin Specific Peptidase 21
WHO: World Health Organization
WIPI2: WD Repeat Domain, Phosphoinositide Interacting 2
XIAP: X-linked IAP
